# Comparison of ultrasound-guided and palpation-inserted peripheral venous cannula in -patients before primary hip or knee arthroplasty: study protocol for a randomized controlled trial

**DOI:** 10.1186/s13063-023-07459-x

**Published:** 2023-07-21

**Authors:** Jakub Hlasny, Roman Alberty, Marian Hlavac, Ivan Grgac, Michael Teodor Grey, Michal Venglarcik

**Affiliations:** 1 Department of Anaesthesia and Intensive Care, Slovak Medical University, F. D. Roosevelt University Hospital, Banska Bystrica, Slovakia; 2 Department of Biology & Ecology, Faculty of Natural Sciences, Matej Bel University, Banska Bystrica, Slovakia; 3 Orthopedic Department, Slovak Medical University, F. D. Roosevelt University Hospital, Banska Bystrica, Slovakia; 4 Institute of Anatomy, Faculty of Medicine, Comenius University Bratislava, Bratislava, Slovakia

**Keywords:** Vein cannulation, Peripheral venous access, Long cannula, Ultrasound-guided vascular access, Randomized trial

## Abstract

**Background:**

More than 2 billion peripheral vascular cannulas are introduced globally each year. It is the most frequently performed invasive procedure in medicine worldwide. There is a group of patients with difficult intravenous access (DIVA). In experts’ hands, ultrasound-guided vascular access appears to be a significantly better method. Investigators hypothesize that UGVA is superior also in short-term patency of cannula and even for blood draw through cannula. Repeated cannula pricks in the operating room setting not only puts a lot of stress on the patient and medical staff, but they also waste OR time.

**Methods:**

This investigator-initiated prospective randomized monocentric controlled trial is designed to randomly allocate 200 patients undergoing elective primary total joint arthroplasty of hip or knee to one of two groups as follows: Group C (control group) – peripheral venous cannula insertion by palpation or Group USG (intervention) – cannula insertion by ultrasound-guided vascular access. Our primary endpoint is to compare the number of attempts for ultrasound-guided insertion of the peripheral venous cannula with common palpation insertion of the peripheral venous cannula in overweight/obese patients (BMI ≥ 25). The secondary endpoint is a failure rate of the peripheral venous cannula to administer intravenous therapy up to 5 days postoperatively. Tertiary endpoints include a portion of long PVCs that are able to ensure blood draw up to 5 days postoperatively, time needed to insert PVC in each group, number of needle tip redirections in both groups, and reinsertion of PVC needed in both groups for any reason.

**Discussion:**

This study is pragmatic and is looking for clinically relevant data. After completion, it will answer the question of whether it is clinically relevant to use ultrasound-guided vascular access in the context of not only short-term benefit of insertion, but also up to 5 days after insertion. Also, if this method can ensure blood draw through a peripheral vein cannula, it can save resources in the perioperative period — valuable especially considering the ongoing shortage of medical staff worldwide. If this hypothesis is confirmed, this finding could contribute to more widespread implementation of ultrasound-guided peripheral vascular access in the perioperative period.

**Trial registration:**

ClinicalTrials.gov NCT05156008. Registered on 13.12.2021.

## Administrative information

Note: the numbers in curly brackets in this protocol refer to SPIRIT checklist item numbers. The order of the items has been modified to group similar items (see http://www.equator-network.org/reporting-guidelines/spirit-2013-statement-defining-standard-protocol-items-for-clinical-trials/).

**Table:** 

Title {1}	Comparison of ultrasound-guided and palpation-inserted peripheral venous cannula in patients before primary hip or knee arthroplasty: study protocol for a randomized controlled trial
Trial registration {2a and 2b}.	ClinicalTrials.gov, under CT number: NCT05156008
Protocol version {3}	version 4.0 (Feb. 16, 2022) of the original protocol
Funding {4}	No funding
Author details {5a}	^1^Department of Anaesthesia and Intensive Care, Slovak Medical University, F. D. Roosevelt University Hospital, Banska Bystrica, Slovakia ^2^Department of Biology & Ecology, Faculty of Natural Sciences, Matej Bel University, Banska Bystrica, Slovakia ^3^Institute of Anatomy, Faculty of Medicine, Comenius University Bratislava, Slovakia ^4^ Orthopedic Department, Slovak Medical University, F. D. Roosevelt University Hospital, Banska Bystrica, Slovakia
Name and contact information for the trial sponsor {5b}	F. D. Roosevelt University Hospital Banska Bystrica, Slovakia, Nám. L. Svobodu 1, Banska Bysrica, Slovakia.
Role of sponsor {5c}	Legal responsibility, provides facilities and medical staff.

## Introduction

### Background and rationale {6a}

**Fig. 1:**
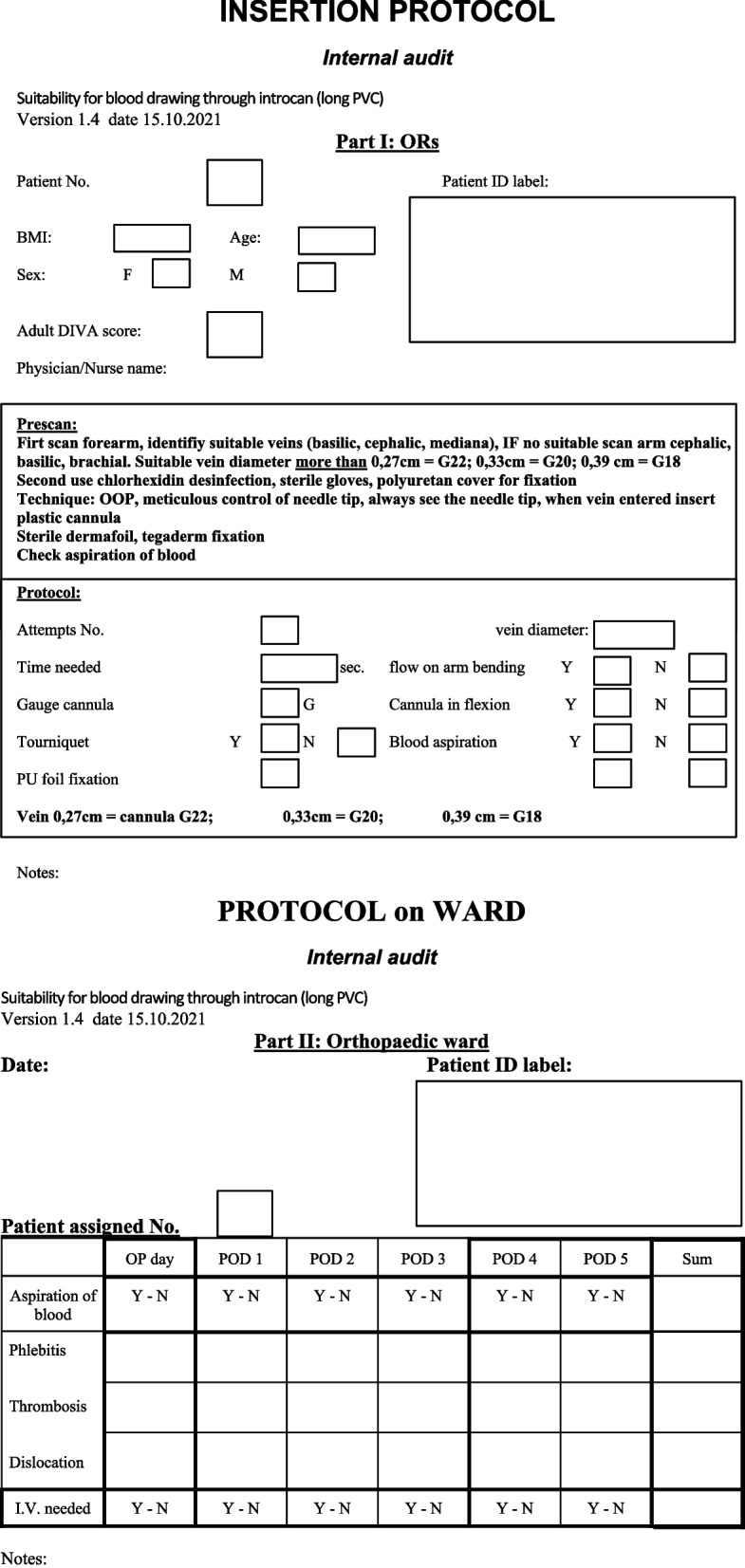
Insertion manual

There is a group of patients with difficult intravenous access (DIVA). In experts’ hands, ultrasound-guided vascular access appears to be a significantly better method. Investigators hypothesize that UGVA is superior also in short-term patency of cannula for up to 5 days and even for blood draw through cannula. In overweight/obese orthopedic patients the DIVA subpopulation can reach up to 50% [1, 2]. Ultrasound seems to facilitate vascular access in patients with higher BMI [3]. The classic landmark technique becomes very difficult in the higher BMI ranges, whereas ultrasound is mostly unaffected by BMI [4]. In our hospital, we perform over 1.000 joint arthroplasties annually. In a small internal hospital audit (*n* = 34) we found out that 60% of inserted cannulas that adhered to strict insertion protocol (Fig. 1) under ultrasound, ensured blood draw on POD (post operative day) 1 and 2. If we can insert a low-cost long PVC that can last up to 5 days and allows for blood drawing, it will ease the perioperative period for DIVA patients and medical staff as well. To the best of our knowledge, no randomized controlled trials have yet been performed to study the effect of ultrasound-guided vascular access of long PVCs in orthopedic overweight patients with focus on i.v. access patency up to 5 days and the ability to draw blood through PVC. Ultrasound can reduce the number of attempts and complications as well, but only in the experienced hands of a fully trained UGVA specialist. Repeated cannula insertions not only put a lot of stress on patients and medical staff. They also waste OR (operating room) time and use up the veins of the patient, which may never heal [5] and this makes vein punctures even more difficult in the future.

### Objectives {7}

The primary objective of this investigator-initiated prospective randomized controlled trial is to compare the number of attempts for ultrasound-guided insertion of PVC with common palpation insertion of PVC in overweight/obese patients. The secondary objective is the comparison of long PVCs and “regular” short PVCs in terms of their ability to administer intravenous therapy through the cannula up to 5 days postoperatively. Tertiary objectives are a portion of long PVCs that are able to ensure blood draw up to 5 days postoperatively, time needed to insert PVC in each group, number of needle tip redirections in both groups, and reinsertion of PVC needed in both groups for any reason.

### Trial design {8}

This investigator-initiated prospective randomized parallel groups monocentric controlled trial is designed to enroll 200 patients. There is no blinding because cannulas are easy to distinguish.

## Methods: participants, interventions, and outcomes

### Study setting {9}

This study is conducted in a single center, in the clinical settings of the Orthopaedic Department of F. D. Roosevelt University Hospital and Operating Rooms by medical staff of the Anesthesia and Intensive Care Department of F. D. Roosevelt University Hospital.

### Eligibility criteria {10}

Inclusion criteria are (1) age group of 18–75 years, (2) body mass index > 25, and (3) primary hip or knee arthroplasty. Exclusion criteria are as follows: (1) reoperation of arthroplasty, (2) mental disorder, (3) sepsis, (4) protocol non-compliance, (5) pregnancy, and (6) patient refusal or no informed consent or both. Interventions are performed by anesthetists trained in UGVA.

### Who will take informed consent? {26a}

Patients scheduled for elective primary total joint arthroplasty of hip or knee surgery in F. D. Roosevelt University Hospital in Banska Bystrica, Slovakia will be asked for written informed consent by a member of the Anesthesiology or Orthopedics Department during preoperative assessment.

### Additional consent provisions for collection and use of participant data and biological specimens {26b}

Not applicable, we do not want to use data in another study. No specimens are collected.

## Interventions

### Explanation for the choice of comparators {6b}

Classic palpation approach for vascular access is challenging in DIVA patients. Up to 50% of orthopedic patients are DIVA [1, 2] and orthopedic surgeries are on the rise in developed countries. Medical staff can site venous cannula only to veins they can see. Therefore, the cannula is often in a suboptimal place of flexion of the arm in the wrist or elbow which are associated with complications [6, 7] and higher failure rates up to 63% [8].

Ultrasound enables us to place the cannula in an ideal place, in terms of the complications mentioned above. In addition, we can measure the diameter of the vein and use cannula width that will obscure only one third of the internal lumen. This should allow for blood draw because of undisturbed bloodstream in the vein.

We chose the UGVA approach in comparison to the classic palpation approach in standard clinical settings. This design of study allows to compare the standard of care (insertion of cannula by palpation) to the modern (ultrasound-guided insertion of cannula), technically more challenging but also a more rewarding option.

### Intervention description {11a}

**Fig. 2:**
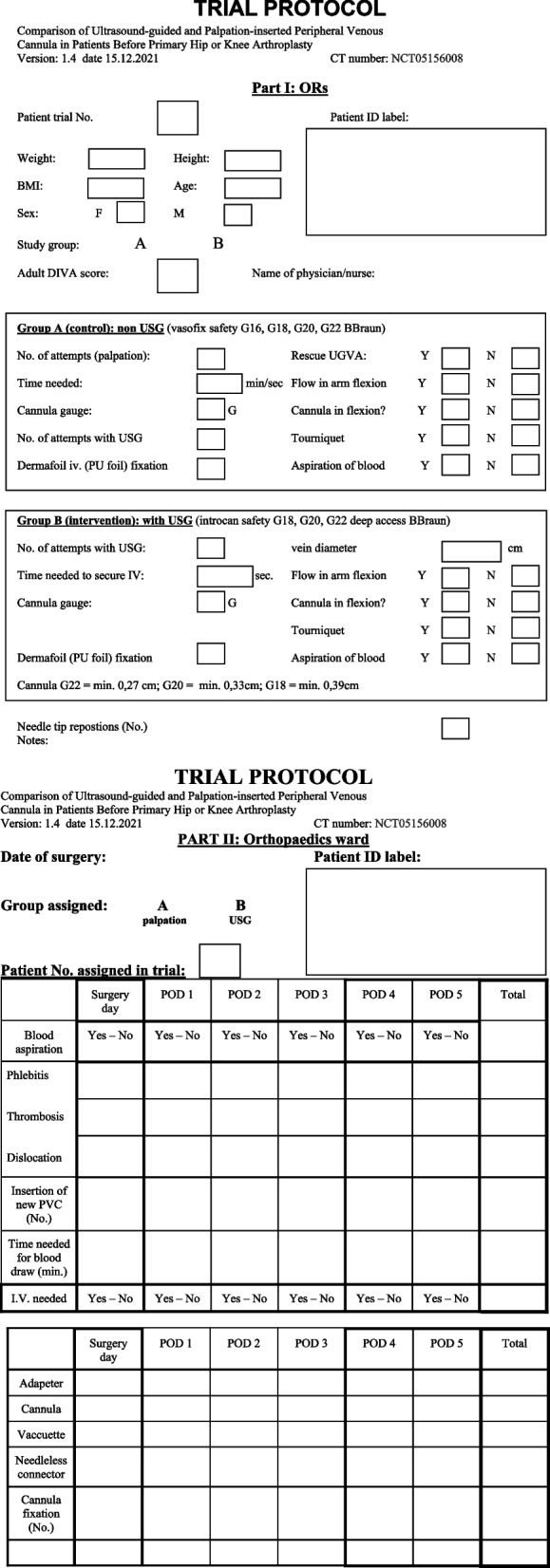
Protocol

All patients in the ultrasound group will receive a prick by an UGVA experienced physician in the block room, preoperatively. The time measurement starts with prescan of the forearm and then, if no suitable vein is found, the anesthetist will prescan the upper arm. Under strict antiseptic precautions, after preparation of the skin with 2% Chlorhexidine (Chlorhexidine, BBraun, Germany) and sterile cover of probe Dermafoil (Dermafoil, Batist, Czech Republic), the introduction of the cannula is carried out by anesthetist. The entire procedure follows our study protocol, using out of plane technique with constant clear visualization of the needle tip, which is slowly advanced all the way into the vein. The procedure may be performed with or without a tourniquet following the insertion manual (Fig. 1). Another researcher makes notes into the study protocol (Fig. 2).

### Criteria for discontinuing or modifying allocated interventions {11b}

The procedure may be performed with or without a tourniquet following the insertion manual (Fig. 1). The method should be discontinued after two unsuccessful attempts and another experienced practitioner should be sought. If such a practitioner is not available it has to be marked in the protocol as “failure” with a note “no experienced practitioner available.” Another researcher makes notes into the study protocol (Fig. 2).

### Strategies to improve adherence to interventions {11c}

Intervention is a single procedure\ event and does not require repetition, therefore adherence is not a feature of intervention delivery.

### Relevant concomitant care permitted or prohibited during the trial {11d}

Prohibited concomitant care is the application of vesicants or irritants infusions through a peripheral vein cannula. Allowed concomitant care is antibiotics therapy which are not vesicants or irritants.

### Provisions for post-trial care {30}

The sponsor has insurance, which is in accordance with the legal requirements in Slovakia. There is no ancillary care. Only common, well-known complications can occur in relation to peripheral vascular access.

### Outcomes {12}

In order to achieve primary and secondary outcomes, the number of attempts, number of cannulas used, tip redirections, time needed to insert the cannula, and proportion of cannulas that requires reinsertion, all this is recorded in the study protocol (Fig. 2) during the procedure. Then researchers will compare both groups to each other. These outcomes are crucial to determine clinical efficacy not just on insertion, but to compare the service life of the cannula inserted. The lifespan of the cannula is related to site of insertion (flexion point), damage to the vessel wall (number of redirections, 1^st^ pass), and proportion of cannula width to vessel diameter. Risks of peripheral vein cannulation are well known and, in general, considered minor.

### Participant timeline {13}

**Table:** 

	Study period					
	Enrolment	List of patient time period	Allocation	Post allocation		Close out
Timepoint	Day − 1	Day – X	0	Day 1	Day 2–4	Day 5
Enrolment:						
Eligibility screen	X					
Informed consent	X					
Randomization		X				
Allocation			X			
Interventions:						
Group C				X		
Group USG				X		
Assessments:						
Baseline variables				X		
Primary outcome				X		
Secondary outcome					X	X

### Sample size {14}

The estimate for the final sample size was based on the expected drop-out rate and the objective and design of the study. This trial assumes a comparison of the effect on outcome variables in peripheral vein insertion in the USG group and palpation-inserted peripheral venous cannula as the control group. In overall, we hypothesize that it will take fewer attempts to insert the cannula into a vein with the USG technique (USG: 1 vs. C: 1–3), corresponding to a reduction in time of ≥ 3 min (clinically relevant indicator) for successful cannulation in the USG group. When calculating the sample size, based on our experience and literature data, we assumed a 20% risk of complications inserting the cannula into a vein in the standard way, 5% as a probability of a Type I error (α < 0.05), and the power of the test 90% (β = 0.10). Theoretically, we also accepted a 5% risk failure of a first insertion of a cannula into a vein in the USG group. The sample size calculation was based on the result of the risk ratio (RR) between groups as % of complications (unsuccessful prick) with cannula insertion into the peripheral veins [9]. The adequate sample size of 162 patients was calculated as described previously [10], increased by 20% for the case unforeseeable circumstances (technical error, data loss, poor patient cooperation, etc.) and rounded ≅ 200.

### Recruitment {15}

The study will include all patients who are indicated for surgery at the time of the registration period and who have met the agreed inclusion criteria. Under the sponsor’s supervision, investigators initiate patient’s enrolment. In our institution, 1.000 arthroplasties are performed annually. The intervention is low cost and necessary to conduct surgery.

## Assignment of interventions: allocation

### Sequence generation {16a}

A random sequence of numbers is generated using SPSS software v. 20.0 (IBM SPSS, Armonk, NY), and this is translated to a series of opaque envelopes which will be used to allocate eligible participants to groups once they have satisfied the inclusion criteria. On the day of surgery, in the block room, the anesthetist will open the envelope and find out the allocation of the patient. According to patient allocation, medical staff will perform the procedure in accordance with the study protocol. This procedure will be repeated until the target sample size is reached.

### Concealment mechanism {16b}

Only one employee working with randomization has knowledge about patient allocation. Each patient allocation is in a sealed opaque envelope. On the day of surgery, in the block room, the anesthetist will open the envelope and find out the allocation of the patient. According to patient allocation, medical staff will perform the procedure in accordance with the study protocol.

### Implementation {16c}

Allocation sequence will be generated by SPSS. Participant enrollment is up to the anesthetists and orthopedic surgeons. Allocation is not concealed and will be revealed to both the patient and the researcher upon randomization.

## Assignment of interventions: blinding

### Who will be blinded {17a}

It is almost impossible to blind care providers or patients because of different appearances of cannulas and different fixation of devices. There is no blinding.

### Procedure for unblinding if needed {17b}

Not applicable. There is no blinding.

## Data collection and management

### Plans for assessment and collection of outcomes {18a}

Written protocol (in paper) provides systematic measurements that assessor has to collect. All assessors will attend compulsory training how to carry out every step in the study. All study-related documents are on cloud that can be accessed by study personnel.

### Plans to promote participant retention and complete follow-up {18b}

Participants are studied only up to 5 days or discharge from the hospital, whichever comes first. The study doesn’t require any extra actions or active collaboration from patients. If the patient deviates from intervention protocols, we will use data of the patient up to the timepoint of deviation if eligible; if not, the patient will be excluded. Exclusion probability is low due to the short period of observation and low to none patient active collaboration.

### Data management {19}

All data is recorded on paper (study protocol) and stored in the anesthetic office in a study case file with limited access. Every day will researchers collect these protocols. Study protocols are then entered into a spreadsheet using Excel (Microsoft Office, 2021, Washington, USA). Double data entry technique will be used with two different researchers or in two different sessions by a single researcher if second is not available. These databases are compared by “IF command in Excel spreadsheet algorithms. After data validity analysis are spreadsheets ready for statistical analysis using SPSS software and Microsoft Excel software.

### Confidentiality {27}

Every patient will get a numerical code instead of his real name on enrolment. Only these codes will be used throughout the study. All emails exchanged between researchers including patients or study data have to use hospital servers only. In addition, every file will be password protected. Hospital standards for data management will be used. During assessors training, there will be time allocated for data management.

### Plans for collection, laboratory evaluation, and storage of biological specimens for genetic or molecular analysis in this trial/future use {33}

Not applicable. No samples were collected.

## Statistical methods

### Statistical methods for primary and secondary outcomes {20a}

The primary and secondary outcomes will be evaluated using the *χ*^2^ test in terms of the difference in approaches of cannula insertion between groups in the number of punctures and the failure rate of the cannulas (failure rate is defined as the proportion of cannulas requiring reinsertion within 5 days after insertion). RR and 95% confidence interval of successful venous cannulation will be also calculated. The primary endpoint is the number of attempts to successful cannulation determined by the number of skin punctures. More attempts are associated with complications. As a secondary endpoint, the failure rate is calculated from the ratio of cannulas that did not fail to cannulas that failed for any reason in each group, up to the first 5 days postoperatively. This data is obtained from the patient protocol (Fig. 2). Primary and secondary outcomes will be reported as frequencies and percentages. For our tertiary outcome, to compare differences between the intervention groups (time required for cannula insertion, number of cannulas used, A-DIVA scoring system, etc.), *t*-test for independent groups or Mann–Whitney U test will be used, if data are not normally distributed. The effect of the intervention on outcome variables will be determined by using paired *t*-test or Wilcoxon signed-rank test for data obtained at baseline and for 5 days of the study. Other endpoints include portion of long PVCs that are able to ensure blood draw up to 5 days postoperatively, time needed to insert PVC in each group, reinsertion of PVC needed in both groups for any reason. All this data is recorded in the study protocol.

### Interim analyses {21b}

Not applicable as no interim analyses are planned.

### Methods for additional analyses (e.g., subgroup analyses) {20b}

When studying with several groups, we will combine these groups appropriately; in other words, we will analyze different intervention versus control subgroups and create simple pairwise comparisons using the same statistical analysis methods that have already been described for categorical or metric (continuous) quantities in the “Data collection and statistical methods” section.

### Methods in analysis to handle protocol non-adherence and any statistical methods to handle missing data {20c}

Not all processes set up in the study deviate from the design of the randomized controlled clinical trial. When analyzing data, if any qualitative or metric data is found, we will first contact a team member who records or measures the data to supplement the missing data, if at all possible, while analyzing the reasons why the data is missing. We will use all available SPSS applications to check/analyze data completeness. In addition, SPSS statistical programs can evaluate and process incomplete data sets correctly (each analysis uses only cases without missing values for all variables and for all analyses). Therefore, we will not artificially attribute any missing values and we will take into account the missing data in the interpretation of the results. Patients with a large amount of missing data will be excluded from the final statistical analysis.

### Plans to give access to the full protocol, participant-level data and statistical code {31c}

The datasets used and/or analyzed during the current study can be made available by the corresponding author upon reasonable request and in agreement with the research collaboration and hospital data transfer guidelines.

## Oversight and monitoring

### Composition of the coordinating center and trial steering committee {5d}

This is a monocenter study designed, performed, and coordinated in the FDR University Hospital in Banska Bystrica, Slovakia. Day-to-day support for the trial is provided by: Principle investigator: takes supervision of the trial and medical responsibility of the patients. Data manager: organizes data capture, safeguards quality and data. Study coordinator: trial registration, coordinates study visits, annual safety reports. Study physician: identifies potential recruits, takes informed consent, ensures follow-up according to protocol. The study team meets once per month or more often if situation demands that. There is no trial steering committee or stakeholder and public involvement group. Online or in person as situation demands.

### Composition of the data monitoring committee, its role and reporting structure {21a}

DMC is not needed. this is not a blinded study, there is no DMC required to protect blinding of the researchers and physicians.

### Adverse event reporting and harms {22}

All adverse events reported by the subject or observed by the investigators will be recorded. The causality to the study treatment event will be recorded. Investigators have to report any adverse events to ethical committee. We use hospital email addresses every employee has on this purpose. All adverse events are collected by the principal investigator.

### Frequency and plans for auditing trial conduct {23}

There is authorized personnel for auditing trial conduct, the process is independent from the sponsor Also Health Care authorities can take place in auditing like Slovak Healthcare Surveillance Authority.

### Plans for communicating important protocol amendments to relevant parties (e.g. trial participants, ethical committees) {25}

If anything, important changes in the study protocol we will reflect that change in the clinicaltrials.gov registry and notify the ethical committee.

### Dissemination plans {31a}

The study will be published in a peer-reviewed journal.

## Discussion

Ultrasound-guided vascular access is an essential skill in modern anesthesia practice. It is recognized also by medical faculties; therefore, in some countries, they include this skill into student’s curriculum. In the beginning of training, it is time-consuming although as the operator gets enough practice whole insertion process takes only seconds. This is the biggest challenge. To ensure that there is always a physician/nurse that is proficient in UGVA.

### Limitations

In order to obtain relevant data, it is necessary to ensure vascular access in both groups by experienced and professionally trained staff. Otherwise, there might be misleading data through the whole study.

DIVA is a widespread problem in many patient groups. We chose orthopedic patients scheduled for total hip- or knee- arthroplasty due to a relatively similar patient population, high patient turnover, higher rates of obesity (which is an inclusion criterion), and most importantly, the electivity of the procedure.

The difference between the types of needle/cannula used in the classic method and the USG method reflects both the different nature of the veins being cannulated (superficial vs. deep), as well as the clinical practice in our institution.

### Strenghts

“Classical” Vasofix needles used in the control group are widely and routinely used by our staff when cannulating veins preoperatively. Deep Introcan needles are in turn used in sonography due to the longer length of the needle required to reach the deeper veins in the forearm and upper arm. Thus, we are comparing the gold standard of the classic method with the gold standard of the USG method. If we would use the same needle in both groups, one group would necessarily receive inferior care – biasing our results and potentially damaging our patients. Study is also focused on not just vascular access obtaining but also on vascular access durability in relation to UGVA. UGVA allows to choose better place to site peripheral vein cannula in comparison with palpation technique which might translate in to lower complication rates [11]. All of these measurements have a great impact on how long this low-cost cannula lasts in DIVA patients [12].

The choice of our outcomes is a comprehensive list of all the potential benefits of USG cannulation: reducing the number of patient pricks for needle insertions and blood draws — increasing satisfaction and reducing complications; reducing time spent in the OR to facilitate increased patient turnover; using less material and saving manpower — making it an economical procedure. 

## Trial status

This document is based on version 4 (Jan. 16, 2022) of the original protocol. We anticipate randomly assigning the first patient on August 1^st^, 2023, and plan to complete the study in July 2024.

## Data Availability

All data will be publicly available on ClinicalTrials.gov.

## Abbreviations

USG: Ultrasound guided
UGVA: Ultrasound-guided vascular access
DIVA: Difficult intravenous access
